# Diversity and Seasonal Dynamics of Ticks on Ring-Tailed Coatis *Nasua nasua* (Carnivora: Procyonidae) in Two Urban Areas from Midwestern Brazil

**DOI:** 10.3390/ani12030293

**Published:** 2022-01-25

**Authors:** Livia Perles, Thiago Fernandes Martins, Wanessa Teixeira Gomes Barreto, Gabriel Carvalho de Macedo, Heitor Miraglia Herrera, Luis Antônio Mathias, Marcelo Bahia Labruna, Darci Moraes Barros-Battesti, Rosangela Zacarias Machado, Marcos Rogério André

**Affiliations:** 1 Laboratory of Imunopharasitology, Department of Pathology, Reproduction and One Health, School of Agricultural and Veterinarian Sciences, São Paulo State University (Unesp), Jaboticabal 14884-900, SP, Brazil; liviaperlesvet@gmail.com (L.P.); barros.battesti@gmail.com (D.M.B.-B.); rzacariasmachado@gmail.com (R.Z.M.); 2 Department of Preventive Veterinary Medicine and Animal Health, School of Veterinary Medicine and Animal Science, University of São Paulo, São Paulo 05319-000, SP, Brazil; thiagodogo@hotmail.com (T.F.M.); labruna@usp.br (M.B.L.); 3 Department of Specialized Laboratories, Superintendence for Endemic Disease Control, State Health Secretariat, São Paulo 05319-000, SP, Brazil; 4 Post Graduation Program of Ecology and Conservation, Mato Grosso do Sul Federal University, Campo Grande 13471-410, MS, Brazil; wanessatgbarreto@gmail.com; 5 Laboratory of Parasitic Biology, Environmental Sciences and Farming Sustainability, Dom Bosco Catholic University, Campo Grande 13471-410, MS, Brazil; carvalhodemacedo@gmail.com (G.C.d.M.); herrera@ucdb.br (H.M.H.); 6 Department of Pathology, Reproduction and One Health, School of Agricultural and Veterinarian Sciences, São Paulo State University (Unesp), Jaboticabal 14884-900, SP, Brazil; la.mathias@unesp.br

**Keywords:** ectoparasites, *Amblyomma sculptum*, *Amblyomma dubitatum*, *Amblyomma ovale*

## Abstract

**Simple Summary:**

The knowledge of the dynamics of ticks in wild animals is essential for surveillance of tick-borne diseases. Coatis (*Nasua nasua*) are mammals that easily adapt to anthropized areas, favoring close contact with domestic animals and humans, favoring the exchange of ticks and tick-borne agents. The present study aimed to investigate the tick diversity on coatis from forest urban areas of midwestern Brazil, as well as the dynamics of ticks during the seasons of the year and the correlation between tick species and gender and age of the sampled coatis. Three tick species were identified parasitizing coatis from forested urban fragments, namely *A. dubitatum* nymphs, *A. sculptum* adults and nymphs, and *A. ovale* adults. After analyzing the obtained results, it is likely that coatis from anthropized areas present tick species diversity lower than those from natural landscapes. The mean intensity and prevalence of *Amblyomma* larvae and nymphs is similar among males and females as well as in immature and mature animals, which might reflect the gregarious behavior of coatis, since adult males live together with females and offspring outside and inside the mating season, forming large groups of individuals.

**Abstract:**

Understanding the diversity and ecology of ectoparasites in wild animals is essential for surveillance of vector-borne diseases. Coatis (*Nasua nasua*) easily adapt to anthropized areas, favoring close contact with domestic animals and humans, with the possibility of exchange of ectoparasites and pathogens. The present study aimed to identify the diversity of ticks parasitizing coatis from forest urban areas of midwestern Brazil, to evaluate the seasonal dynamics of ticks during the seasons of the year, and to assess the correlation between tick species and gender and age of the sampled coatis. For this purpose, 103 coatis were captured in two Conservation areas, both located in Campo Grande city, Mato Grosso do Sul state, Midwestern Brazil. The animals’ entire body was inspected for the presence of ectoparasites, and ticks were removed for taxonomic identification. In total, 168 captures were performed in both areas during the observational study considering the first capture and recaptures. In total, 2242 ticks were collected: 838 *Amblyomma* larvae, 1241 *A. sculptum* nymphs, and 150 *A. dubitatum* nymphs. Thirteen adult ticks were identified as three males and five females of *A. sculptum* and two males and three females of *A. ovale.* While a quantity of *Amblyomma* larvae was observed in the first months of the year (January, April and May), *Amblyomma* nymphs showed a higher quantity during the months of July, August, October and November. No statistical difference was observed when comparing mean intensity and prevalence of *Amblyomma* larvae, nymphs of *A. sculptum* and *A. dubitatum* between the two sampled areas, males vs. females and immature vs. mature animals. In conclusion, three tick species were identified parasitizing coatis from forested urban fragments in midwestern Brazil, namely *A. dubitatum* nymphs, *A. sculptum* adults and nymphs, and *A. ovale* adults. Coatis from anthropized areas seem to present tick species diversity lower than those from natural areas. The lack of statistical difference regarding mean intensity and prevalence of *Amblyomma* larvae and nymphs between males vs. females and immature vs. mature animals might have reflected the gregarious behavior of coatis, since adult males live together with females and offspring outside and inside the mating season, forming large groups of individuals.

## 1. Introduction

Ring-tailed coatis (*Nasua nasua*) are medium-sized animals belonging to the order Carnivora and the family Procyonidae [1]. Such mammals have a wide geographic distribution in South America, and easily adapt to different environments, especially urbanized areas [2]. This plasticity favors close contact with domestic animals and humans, with the possibility of exchange of ectoparasites and associated pathogens [3]. Carnivores are considered the first source of human infections with zoonotic agents, which include tick-borne pathogens [4,5,6]. Tick-borne pathogens have already been detected in coatis and associated ectoparasites from Brazil [7,8,9,10]. *Theileria* sp., *Anaplasma bovis*, *Anaplasma* sp. closely related to *A. phagocytophilum Ehrlichia* sp., and hemoplasmas were detected in coatis from Pantanal wetland, Mato Grosso do sul state, central-western Brazil [7,8,9]. Recently, *Rickettsia bellii* and *Rickettsia amblyommatis* were detected in *Amblyomma ovale* and *Amblyomma coelebs*, respectively, from coatis sampled in Iguazu National Park, Iguazu Falls, Paraná state, southern Brazil [10]. Therefore, the constant surveillance of species, diversity and ecology of ectoparasites in wild animals is essential for monitoring vector-borne diseases.

The Brazilian tick fauna is currently comprised of 75 species, including 24 Argasidae species and 51 Ixodidae species [11,12,13,14]. Within the Ixodidae family, the genus *Amblyomma* is the most representative, with 33 species described [11,12,15]. The *Amblyomma* genus is generally composed of three-host tick species, except for *Amblyomma rotundatum* that may present a two-host behavior in snakes and chelonians [16,17] and three-host behavior when fed on frogs [18]. Many tick species present a life cycle of one generation per year with most adults during warmer months, such as *Amblyomma* sp. [19,20,21,22,23]. Depending on tick species, they may present questing or nidicolous behavior. *Amblyomma* spp. are questing ticks, which climb on to vegetation to wait for the passing host (ambush ticks) [24,25]. *Amblyomma sculptum* also exhibits a hunter behavior, where some specimens can emerge from their refuges and run across the ground to find the host. This tick species has a highly aggressive behavior, and it is considered a major human biting tick species in Brazil [23]. Even though *Amblyomma ovale* is also considered a questing tick, a specific population of this tick species exhibited a nidicolous behavior, with all three stages feeding on dogs in an anthropized area in Peruibe in southeastern Brazil [26].

Previous studies assessing the diversity of ectoparasites on coatis from Brazil have showed infestation by *Amblyomma brasiliense* and *Amblyomma ovale* in Paraná state [27]; *A. ovale*, *Amblyomma parkeri*, *A. brasiliense, Amblyomma* spp., *Rhipicephalus (Boophilus) microplus, Rhipicephalus sanguineus* sensu lato (both *Rhipicephalus* from domestic animals) and *Amblyomma sculptum* in Mato Grosso [3]; São Paulo [28,29,30], Paraíba [31] and Minas Gerais states [28,32] and *A. rotundatum* in Pará state. In a study carried out in the Brazilian Pantanal, Mato Grosso do Sul state, coatis were found to be infested by *A. sculptum*, *Amblyomma parvum*, *A. ovale*, *R. (B.) microplus* and *Amblyomma* spp. [7,8,9]. In the state of São Paulo, in the cities of Botucatu and Palmital, infestations by *Amblyomma dubitatum*, *A. ovale*, *A. sculptum* and *R. sanguineus* s. l. were reported [33]. Gaining knowledge on the diversity and seasonal dynamics of tick species that parasitize free-ranging animals living in urban forest fragments is important to the surveillance of tick-borne diseases in order to establish control measures and also to support the conservation of species of wild animals. Herein, we hypothesized that the diversity of tick species found in anthropized areas is lower than in natural landscapes. The aims of the present study were: (1) to identify the ticks found parasitizing coatis from urban areas of midwestern Brazil; (2) to evaluate the seasonal dynamics of ticks found parasitizing coatis; and (3) to assess the association between tick species and gender and age of the sampled coatis.

## 2. Materials and Methods

### 2.1. Sampling Area

**Figure 1:**
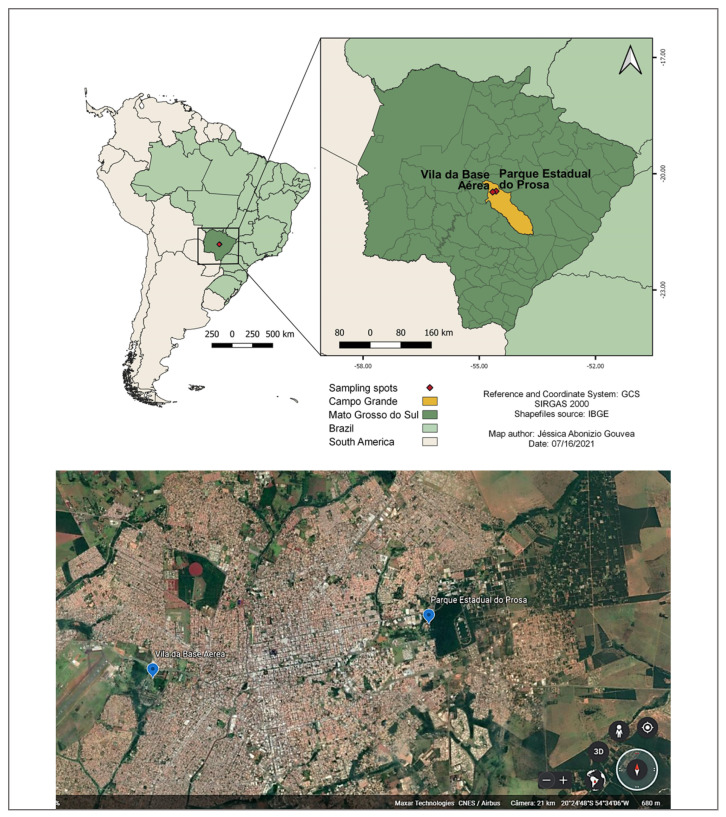
Map showing the state of Mato Grosso do Sul (dark green), with Campo Grande city highlighted in yellow. Source: QGIS Development Team, 2020. QGIS Geographic Information System. Open-Source Geospatial Foundation Project. http://qgis.osgeo.org Accessed on 10 November 2021.

Ring-tailed coatis (*N. nasua*) were captured in two Conservation areas, both located in Campo Grande city, Mato Grosso do Sul state, in midwestern Brazil. The phytophysiognomy of Campo Grande is Cerrado, characterized by a tropical savanna composed by areas of grassland to a nearly closed canopy of medium height trees overlying grass. Campo Grande has a tropical savanna climate, with semi-humid, hot summers, and notably seasonal, with marked dry weather from April to October and rainy weather from November to March [34]. The two samplings’ spots in Campo Grande were “Parque Estadual do Prosa” (PEP) (−20.44987, −54.56529) and “Vila da Base Aérea” (VBA) (−20.47163, −54.65405) (Figure 1).

PEP is a state National Conservation Park 134 ha in size, representing one of the last remnants of the Cerrado biome within the urban perimeter. It preserves regional species of fauna and flora threatened by extinction. The area has become a tourist spot for visitors from cities in the state of Mato Grosso do Sul as well as from different regions of Brazil, so there is a high daily circulation of people (approximately 2000 visitors attend the PEP during the week, and on weekends, it can exceed 6000 users) [34]. It is an important refuge for wildlife, where many species of mammals can be found, such as ring-tailed coatis (*N. nasua*) [34,35], anteaters (*Myrmecophaga tridactyla* and *Tamandua tetradactyla*) [34], capybaras (*Hydrochoerus hydrochaeris*) [34] opossums (*Didelphis albiventris*) [36,37,38], bats (e.g., *Artibeus* sp. and *Myotis nigricans* [39], birds (e.g., Cuculidae and Psittacidae) and reptiles (*Tupinambis* spp.) [34]. Also, this park has a wild animal rehabilitation center, which usually receives free-living animals belonging to several species (available at: https://www.imasul.ms.gov.br/gestao-de-unidades-de-conservacao/unidades-de-conservacao-estaduais/parque-estadual-do-prosa-pep/ Accessed on 19 November 2021). In addition to wildlife, domestic cats and dogs have been seen on site. Two density studies were performed at PEP with coatis, where 33.7 individuals/km^2^ were estimated in 2009, equivalent to 120 coatis in the study area [13] and 11.2 individuals/Km^2^ in 2021 (30 coatis in the study area) [14].

VBA is a Brazilian Air Force base with an area of approximately 484 ha that presents fragments of Cerrado forest, but no visitors are allowed. It’s a residential area, surrounded by three forest fragments and one area used for military training. At least 730 people and their domestic animals inhabited the residential complex. The houses are not fenced, and they all had a trash can at the front. Coatis have access to the outside of the houses, and they were observed during the captures walking around the houses and also searching for food in the dumpsters. Only one density study was performed at VBA, where 19.4 individuals/km^2^ were estimated, equivalent to 41 coatis in the study area [14]. Differences between both sampling areas are shown in the text and Table 1.

### 2.2. Capture of the Animals

Ring-tailed coatis were captured every three weeks for 10 consecutive days (with intervals of two days—Saturday and Sunday) between March of 2018 and April of 2019. All captures and recaptures were performed by convenience, and dates from recaptures were by chance. Animals were captured using metal traps (1 m × 0.40 m × 0.50 m) placed arbitrarily according to the possibility of human access and availability of shadow, covering most of the PEP and VBA areas. Captured animals were anesthetized with an association of Tiletamine hydrochloride and Zolazepam hydrochloride (Telazol, Zoetis^®^ (Parsippany-Troy Hills, NJ, USA) ±6 mg/kg, intramuscularly) [34]. After chemical restraint, animals were marked with numbered colored earrings and had a microchip implanted in the subcutaneous tissue between the shoulder blades in order to identify them for future recaptures. Animals were measured and the age was estimated according to Olifiers et al. [40]. The animals’ entire body was inspected for the presence of ectoparasites for three minutes. Ticks were removed with the aid of a forceps and stored in 100% alcohol (Merck Ensure^®^, Darmstadt, Germany)-containing RNAse/DNAse free microtubes. Taxonomic identification of ectoparasites was performed with a stereoscopic microscope (Olympus SZX7, Tokyo, Japan) following taxonomic literature [11,41]. While larvae were identified to genus level, nymphs and adults were identified to species level. All the specimens examined were deposited in the Acari Collection of the Instituto Butantan, São Paulo, Brazil (IBSP) (Figures S1–S7).

### 2.3. Statistical Analyses

The prevalence was obtained by calculating number of infested coatis divided by the total of sampled coatis. An analysis of the association between the dichotomous dependent variables was performed (presence/absence of ectoparasites (total ticks—all stages; larvae and nymphs) and categorical independent variables (sampling spot (PEP vs. VBA), gender (female vs. male) and age group (immature vs. mature) to detect variables with significant association (*p* < 0.05) in the X² test using the Epi Info software [42]. Values of odds ratio and superior and inferior confidence interval were obtained. The intensity (range of infestation: minimum and maximum) and mean intensity (MI) (total number of ticks (larvae, nymphs and adults) ÷ number of infested coatis] were assessed. All observations (terms) were defined according to Margolis et al. [43]. For seasonal analyses (months of the year and dry vs. weather season), all data collected was analyzed independently of gender, age or sampling spot. Adult ticks were not included in the statistical analyses due to the small number of specimens sampled. General linear mixed effects models (GLMM) were performed to test if there was an effect of the fixed factors (locality, gender, and age, separately and interactions) in the quantity of ticks to detect factors with significant association (*p* < 0.05) using Glimmix at Statistical Analysis System (SAS). In order to identify seasonal patterns, the number of tick specimens collected in each month were analyzed separately from 2018 and 2019. For statistical analyses regarding sampling spots, gender and age, only the first capture was assessed, since recaptures were performed in different periods of the year (by convenience). For seasonal analyses, all data collected was analyzed independently of gender, age or sampling spot, but data collected from 2018 were analyzed separately from those obtained during 2019. Mean quantity of larvae and nymphs ticks (Total number of collected ticks ÷ number of total sampled coatis) were used to evaluate distribution of sampled ticks during months of the year. Information regarding temperature and precipitation in Campo Grande city during 2018 and 2019 were collected from the governmental website of “Centro de Monitoramento do Tempo e Clima de Mato Grosso do Sul” (https://www.cemtec.ms.gov.br/boletins-meteorologicos/ Accessed on 14 January 2022).

## 3. Results

### 3.1. Sampling Ring-Tailed Coatis

In total, 168 captures were performed in both areas during the observational study, including the first capture and one to three recaptures (69 in PEP and 99 in VBA). In PEP, samples were obtained from 48 different coatis (30 females and 18 males; eight cubs, one subadult, and 39 adults). One capture (without recapture) was performed in 32/48 individuals, two captures in 12/48 coatis (first sampling and one recapture), 3/48 animals were recaptured twice (first sampling and two recaptures), and only 1/48 animals was recaptured three times (first sampling and three recaptures), totaling 69 captures in PEP. In VBA, samples were obtained from 55 different individuals (33 females and 22 males; 10 cubs, seven subadults and 38 adults). One capture was performed in 33/55 individuals (one sampling), two captures were performed in 11/55 coatis (first sampling and one recapture), 4/55 were recaptured twice (first sampling and two recaptures), 5/55 were recaptured three times (first sampling and three recaptures) and 2/55 were recaptured five times (first sampling and five recaptures), totaling 99 captures from VBA.

### 3.2. Ticks Specimens Collected from Ring-Tailed Sampled Coatis

**Figure 2:**
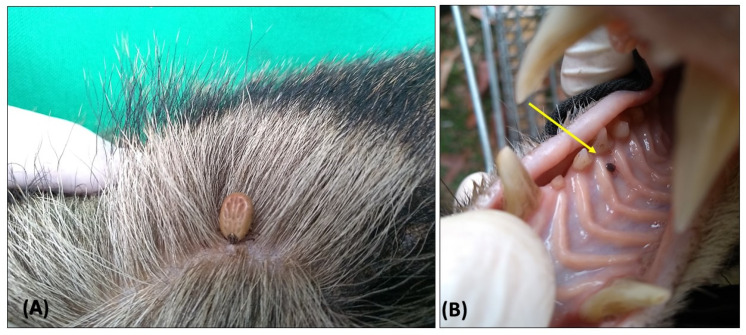
Ticks collected from ring-tailed coatis (*Nasua nasua*) sampled in Campo Grande city, Mato Grosso do Sul state, Brazil. (**A**) Partially engorged female of *Amblyomma ovale* feeding on the dorsum of a coati. (**B**) Nymph of *Amblyomma sculptum* attached on the palate of a coati.

In total, 2242 ticks were collected from coatis in the present study in both areas, including animals at the first capture and in the recaptures (considering 168 captures). Regarding tick species, 838 larvae were identified as *Amblyomma* spp. Among 1391 nymphs, 1241 were identified as *A. sculptum* and 150 to *A. dubitatum*. Co-infestation by both *A. sculptum* and *A. dubitatum* nymphs were observed in 36 animals. Thirteen adults were identified as three males and five females of *A. sculptum* and two males and three females of *A. ovale*. Regarding adult ticks, only one coati (VBA 27) was co-infested, with two adults belonging to different tick species, represented by one male *A. sculptum* and one male *A. ovale*. Eleven coatis were infested by only one adult tick (four coatis with one *A. ovale* each and seven coatis with one *A. sculptum* each). Ticks were collected from all regions of the body of the animals (Figure 2A), either engorged or unengorged, especially in the paws (between fingers), around the eyes and lips, ear tip and genital areas (around foreskin and vulva). Interestingly, one nymph of *A. sculptum* was removed from inside the mouth during physical examination, and the specimen was feeding on the palate (Figure 2B).

Regarding the first capture (used for statistical analyses), 455 larvae of *Amblyomma* spp. were obtained from both areas. The prevalence of infested coatis was 32/48 (66.67%) for PEP vs. 31/55 (56.36%) for VBA (Figure 2). For nymphs, a total of 735 specimens were collected, corresponding to 76 *A. dubitatum* and 659 *A. sculptum*. The prevalence of infested coatis with A. *dubitatum* was 23/48 (47.92%) for PEP vs. 21/55 (38.18%) for VBA; for *A. sculptum* it was 36/48 (75%) for PEP vs. 42/55 (76.36%) for VBA (Figure 2). Co-infestation by both *A. sculptum* and *A. dubitatum* nymphs was observed in 19 animals from PEP and 17 from VBA. Regarding adult ticks, only five specimens were sampled on four coatis during the first capture from VBA. One coati (VBA 27) was co-infested with one *A. sculptum* male and one *A. ovale* male. *Amblyomma ovale* adults were found only at VBA, while *A. sculptum* adults were found in both areas. Adult ticks were not included in the statistical analyses due to the small number of collected specimens.

### 3.3. Gender and Age

**Figure 3:**
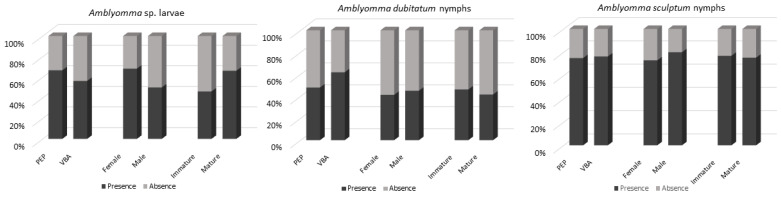
Graphical analyses demonstrating prevalence of coatis infested by *Amblyomma* larvae and nymphs of *A. dubitatum* and *A. sculptum* by percentage.

Taking into account the first capture, 43/63 (68.25%) females and 23/40 (57.5%) males were found parasitized by *Amblyomma* larvae (Figure 3). Considering that adult ticks were collected from only one female and three males, they were not included in the statistical analyses due to the small number of specimens collected. Taking into account the first capture, 12/26 (46%) immature and 51/77 (66%) mature animals were found parasitized by *Amblyomma* larvae (Figure 3). No adult tick was collected from immature animals.

The proportion of coatis infested by *Amblyomma* larvae and nymphs of *A. dubitatum* and *A. sculptum* (evaluated separately) showed no significant statistical difference between the two sampling spots, gender, and age (*p >* 0.05). Values of confidence interval (superior and inferior), *Odds ratio* and *p*-value are showed in Table S1 (Supplementary file).

All descriptive values (total, intensity, and mean intensity) from non-recaptured animals are shown in Table 2. Effects of fixed factors (locality, gender, and age) and their interactions (locality and gender; locality andage, gender and age and locality, genderandage) were not observed on the quantity of ticks (*p* > 0.05—GLMM) (Supplementary file—Table S2). Therefore, we can assume that the prevalence (number of infested coatis ÷ number of sampled coatis) and quantity of ticks (*Amblyomma* sp. larvae, *A. dubitatum* and *A. sculptum* evaluated separately) is not affected by age and gender of the coatis sampled in the present study and did not differ from PEP vs. VBA.

### 3.4. Seasonal Dynamics

**Figure 4:**
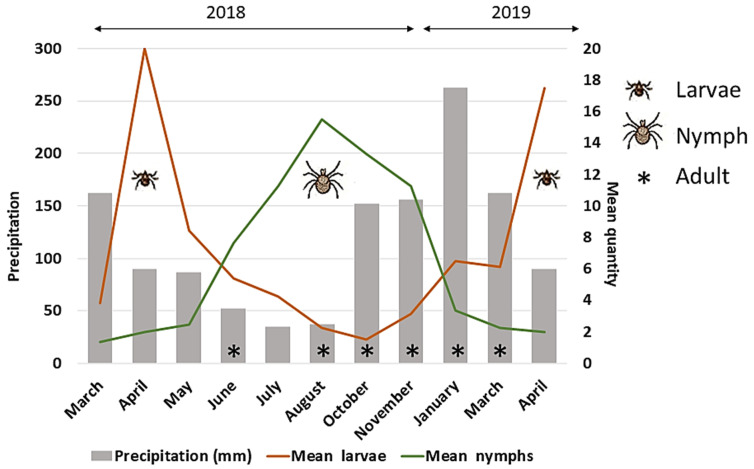
Mean quantity of tick larvae and nymphs collected from ring-tailed coatis (*Nasua nasua*) sampled in Campo Grande city, Mato Grosso do Sul state, Brazil, according to the month of sampling, in the years of 2018 and 2019. Months when adult ticks were collected are showed as (*). Recaptures were included.

Larvae of *Amblyomma* sp. and nymphs of both *A. dubitatum* and *A. sculptum* were sampled in all months of the year, but differences in the mean abundance of infestation were obtained. Regarding *Amblyomma* larvae, a higher mean of infestation was observed in the first months of the year (January, April and May). Nymphs presented opposite results, with higher mean of infestation in the second semester (July, August, October and November) when analyzing *A. sculptum* and *A. dubitatum* together. The thirteen adult ticks were collected in five different months (June 2018—1 *A. ovale*, 1 *A. sculptum*), (August 2018—2 *A. ovale*, 2 *A. sculptum*, 1 *A. ovale*), (October 2018—2 *A. sculptum*, 1 *A. ovale*) (November 2018—1 *A. sculptum*), (January 2019—1 *A. sculptum*), (March 2019—1 *A. sculptum*) (Figure 4; Table 2).

## 4. Discussion

Understanding the diversity and ecology of ectoparasites in wild animals is essential for surveillance of vector-borne diseases. Some changes in natural areas that lead to the absence of predators and the high availability of food can act as factors that lead to adaptation of coatis in anthropized environments, and thus the consequent increase in population densities [32]. Mainly in urban environments, the constant contact between wildlife, domestic animals and humans constitutes risk factors for the emergence of tick-borne diseases [44]. Also, some tick species that may be found in an anthropic environment may present a potential risk to public health, since they can transmit some important pathogens with zoonotic potential.

In the present study, three tick species were identified parasitizing coatis from midwestern Brazil, including *A. dubitatum* nymphs, *A. sculptum* adults and nymphs, and *A. ovale* adults. The dominant species, representing 89% of all collected nymphs was *A. sculptum*. A few specimens of adult *A. ovale* and nymphal *A. dubitatum* were also found, albeit in low numbers. Similar results were found when investigating ticks from wild carnivores (*Lycalopex vetulus, Cerdocyon thous, Chrysocyon brachyurus* and *Puma concolor*) in the Brazilian Cerrado, where *A. sculptum* represented 98.6% of the total number of sampled ticks, mainly in the nymphal stage [44]. The present study is also in agreement with other studies carried out in several regions of Brazil, which also indicated a strong association between *A. sculptum* nymphs and wild carnivores [29,45,46]. *Amblyomma sculptum* is a generalist species, widely distributed in the Brazilian biomes of Cerrado, Pantanal, and degraded areas of the Atlantic forest [44,47,48,49,50]. Due to its adaptive plasticity, this species development is favored in degraded areas under anthropogenic influences [44,49]. In these degraded forest areas, *A. sculptum* is the most common human-parasitizing tick and has significant importance as the principal vector of the deadly Brazilian spotted fever pathogen, *Rickettsia rickettsii* [23,25,26]. Due to hunter and aggressive behavior of this tick species [23], the present report shows the importance of tick species monitoring, since the sampled coatis have access to places with high daily circulation of people (PEP) and close contact with human houses (VBA), making human bites and vector-borne pathogen transmission possible.

The present study includes the second report of *A. dubitatum* nymphs feeding on ring-tailed coatis. This tick species has already been found parasitizing coatis from São Paulo state [33]. All stages of *A. dubitatum* are primarily associated with capybaras (*H. hydrochaeris*), although there are reports of immature and adult stages in other mammals, such as tapirs (*Tapirus terrestris*) [51], crab-eating foxes (*C. thous*) [29], humans [23,52] as well as larvae and nymphs in black-eared opossums (*Didelphis aurita*) [53], white-eared opossums (*Didelphis albiventris*) [36,53], and Cricetidae rodents [54]. Interestingly, an interspecific association between coatis and capybaras was found in the Parque das Nações Unidas [55] located aside the PEP (where the present study was carried out). Rucco et al. [55] observed coatis feeding on ticks attached to capybaras, in a protocooperation between the two mammal species. Also, we observed capybaras resting aside from a coati captured in a trap in November 2021 at PEP (in a new campaign for a co-related study) (Figure S8). We suggest that the proximity of these two mammal species might have been associated with the presence of *A. dubitatum* nymphs on coatis; however, further studies are necessary to understand if this protocooperation can contribute to ticks’ exchange between these two host species.

No statistically significant differences in tick species diversity, mean intensity, or prevalence was observed between coatis from the two sampled areas. PEP is a conserved area surrounded by urban environments and VBA is an anthropized environment surrounded by three forest fragments. Both areas have fluid movement of coatis between urban and wild areas, which may explain the lack of difference found in the present study. Biodiversity of host species in conserved areas, such as mammals, birds and reptiles, tend to be higher and more evenly distributed than in anthropized areas, since habitat loss and fragmentation may be a bias in the host species community, leading to the dominance of a few generalist species, such as coatis [56,57]. Previous studies demonstrated that the abundance of some mammalian hosts has a direct effect on tick species/abundance, since the more the host abundance, the better the chances for ticks finding hosts to complete their life cycle, favoring the increase of a certain tick species population [56,58,59,60]. Although *A. sculptum* is primarily associated with capybaras, this tick species has also been described in a large variety of wild carnivores; indeed, this tick species is considered as a plastic species, adapting easily to anthropized environments. The fragmentation of both sampled areas that may serve to decrease the diversity of other mammal hosts might have led to the low diversity of tick species in the present study.

Interestingly, *A. ovale*, a tick species that is reported in wild carnivores and has already been described parasitizing coatis [29], was found only on coatis sampled in VBA. Unfortunately, no study on host diversity and abundance has been performed at PEP or VBA regarding the presence of wild carnivores, which in turn may help maintaining the *A. ovale* life cycle. Since we have no further information, it is impossible to assume that the occurrence of *A. ovale* in coatis only from VBA was due to a higher number of coatis sampled at VBA (which might have increased the possibility of collecting *A. ovale* adults); alternatively, VBA may have other wild carnivores along their forest fragments. Also, information is scarce regarding ticks parasitizing other hosts in the studied area. Previously, our research group [36] collected nymphs of A. *dubitatum* from *D. albiventris* at PEP.

*Rhipicephalus (Boophilus) microplus* and *Rhipicephalus sanguineus* sensu lato, both species related to domestic animals, were previously collected from free-living coatis in Pantanal, Mato Grosso do sul state [7], and captive coatis from a zoobothanical park from Paraíba state [33], respectively. *Rhipicephalus (Boophilus) microplus* has a worldwide distribution and is considered the most important tick of livestock in the world, due to the vector competence for *Babesia bigemina, Babesia bovis* and *Anaplasma marginale* [61,62]. Brazilian Pantanal is an important area for the cattle industry, where there are reports of wild animals sharing areas with cattle, which may lead to ticks exchange [63]. *Rhipicephalus sanguineus* is also found worldwide and is primarily associated with dogs, although there are reports of infestations of humans and other animals [64,65]. Both reports [7,33] found only one specimen of each *Rhipicephalus* species on coatis, which may be interpreted with caution, since it may be an occasional finding and therefore without epidemiological implications. Although both areas sampled in the present study have circulation of domestic animals (stray cats and dogs), no *R. sanguineus* or *R. (Boophilus) microplus* was collected from coatis.

Understanding the parasite-host relationship is essential for investigating the dynamics of diseases and evolutionary implications of the parasites in their hosts and ecosystems. A commonly observed pattern in this relationship is a higher intensity of pathogens in young animals compared to adult animals [66,67]. Reasons for this difference are not fully understood, and may vary according to the parasite species, host and environment. In the present study, no statistically significant difference was observed when comparing mean intensity and mean abundance of ticks between different ages (immature vs. mature). Taking into account the biology of the coati, it is known that they live in large groups, with cubs, subadults and adults sharing the same environment. Females give births in tree nests, and cubs leave those nests around five to eight weeks after birth [68,69], and then remain as subadults in the same group. We suggest that the absence of statistical differences may be due to the fact that coatis from all ages have similar chances to be in contact with arthropod vectors from the moment the cubs leave their nests. This type of observation may be important for future studies when comparing the chance of a coati from a certain age to be infected by a tick-borne pathogen.

The prevalence and intensity of parasitic infections appear to be higher in males than in females. This fact can be explained by the behavior of some species, in which males, which are more prone to aggressive interactions (to conquer females and territories) and dispersion, would be more likely to have contact with ectoparasites and associated pathogens [70]. However, several studies have found no gender differences in the prevalence or intensity of parasite infection [33,71], and some have even found higher rates of parasite infection in females [72]. No statistically significant differences were observed between genders of coatis. Previous studies suggested that *N. nasua* would have a similar social system to *Nasua narica*. While solitary adult males live apart from the group outside the mating season, females live in groups with their offspring [73,74,75]. This behavior may lead to higher prevalence and intensity of ectoparasites and parasitic infections in males than in females because they tend to disperse in a larger territory [70]. However, studies about dietary patterns and behavior of *N. nasua* showed different features, with adult males observed living together with females and offspring outside of the mating season in five different coati populations in Brazil (Mangabeiras Park, Minas Gerais; Tiete Ecological Park, São Paulo; Nhumirim ranch, Pantanal; *Parque Estadual do Prosa*, Mato Grosso do Sul; and Campeche Island, Santa Catarina) and in Foz do Iguaçu, Argentina, [1,13,34,35,36]. It seems that both in natural [69] and anthropized areas (such as those sampled in the present study) males remain together with the group outside of the mating season. This behavior might explain the lack of statistical differences in the presence of ticks between males and females, since they cohabit the same environment.

## 5. Conclusions

Three tick species were identified parasitizing coatis from midwestern Brazil, namely *A. dubitatum* nymphs, *A. sculptum* adults and nymphs, and *A. ovale* adults. We observed low tick species diversity on coatis from two highly anthropized areas. The lack of statistical difference regarding mean intensity and prevalence of *Amblyomma* larvae and nymphs between males and females and immature and mature animals might have reflected the gregarious behavior of coatis, since adult males live together with females and offspring outside and inside the mating season, forming large groups of individuals.

## Figures and Tables

**Table 1:** Differences between the two urban fragments from Campo Grande city, Mato Grosso do Sul state, central-western Brazil, where coatis (*Nasua nasua*) where sampled between March of 2018 and April of 2019.

Characteristics	Parque Estadual do Prosa (PEP)	Vila da Base Aérea (VBA)
Geographical coordinates	−20.44987, −54.56529	−20.47163, −54.65405
Type of sampling area	National Conservation Park	Brazilian Air Force base
Sampling area size	135 ha	484 ha
Estimated coatis’ density	33.7 individuals/km^2^ in 2009 (120 animals) [13] and 11.2 individuals/km^2^ in 2021 (30 animals) [14]	19.4 individuals/km^2^ (41 animals) [14]
Visitors allowed	Yes	No
Residential area	No	Yes
Presence of domestic cats/dogs	Yes	Yes
Wild animal rehabilitation center	Yes	No

**Table 2:** Total, intensity, and mean intensity of tick specimens collected from ring-tailed coatis (*Nasua nasua*) sampled in Campo Grande city, Mato Grosso do Sul state, Brazil, according to locality, gender and age. Recaptures were not included.

	Variables		*Amblyomma* spp. Larvae	*Amblyomma dubitatum* Nymphs	*Amblyomma sculptum* Nymphs	Adults
PEP vs. VBA	Total in both areas		455 specimens	76 specimens	659 Specimens	5 specimens
	Total by area	VBA ^1^	277	46	384	5
		PEP ^2^	178	30	275	-
	Intensity *	VBA	1–33	1–7	1–34	1–2 ^+^
		PEP	1–31	1–4	1–23	-
	Mean intensity **	VBA	8.93 (277/31)	2.19 (46/21)	9.14 (384/42)	1.1 (11/10)
		PEP	5.56 (178/32)	1.3 (30/23)	7.63 (275/36)	-
Immature vs. Mature Female vs. Male	Total in both genders and ages		735 specimens	659 Specimens	5 specimens	5 specimens
	Total by gender	F ^3^	286	42	458	1
		M ^4^	169	34	201	4
	Total by age	IM ^5^	102	24	134	-
		MAT ^6^	353	52	225	5
	Intensity *	F	1–31	1–7	1–34	1 ^+^
		M	1–32	1–6	1–24	1–2
		IM	1–32	1–6	1–21	-
		MAT	1–31	1–7	1–34	1–2
	Mean intensity **	F	6.65 (286/43)	1.61 (42/26)	9.95 (458/46)	1 (1/1)
		M	8.45 (169/20)	1.88 (34/18)	6.48 (201/31)	1.33 (4/3)
		IM	8.5 (102/12)	2 (24/12)	6.07 (134/20)	-
		MAT	6.92 (353/51)	1.6 (52/32)	9.05 (525/58)	1.25 (5/4)

* Range of individual infestation: minimum and maximum. ** Total number of ticks (larvae, nymphs and adults) ÷ number of infested coatis. ^1^ Vila da Base Aérea. ^2^ Parque Estadual do Prosa. ^3^ Female. ^4^ Male. ^5^ Immature. ^6^ Mature. ^+^ *Amblyomma ovale* was found only on coatis from VBA.

## Data Availability

The raw data generated in this study can be obtained by reasonable request to the corresponding author.

## Supplementary Materials

The following supporting information can be downloaded at: https://www.mdpi.com/article/10.3390/ani12030293/s1, Figure S1: Non-engorged *Amblyomma* spp. larvae collected from a ring-tailed coati (*Nasua nasua*) sampled in Campo Grande city, Mato Grosso do Sul state, central-western Brazil. Figure S2: *Amblyomma sculptum* nymphs collected from ring-tailed coatis (*Nasua nasua*) sampled in Campo Grande city, Mato Grosso do Sul state, central-western Brazil. Figure S3: *Amblyomma dubitatum* nymphs collected from ring-tailed coatis (*Nasua nasua*) sampled in Campo Grande city, Mato Grosso do Sul state, central-western Brazil. Figure S4: *Amblyomma ovale* male collected from a ring-tailed coati (*Nasua nasua*) in Campo Grande city, Mato Grosso do Sul state, central-western Brazil. Figure S5: *Amblyomma ovale* engorged female collected from a ring-tailed coati (*Nasua nasua*) sampled in Campo Grande city, Mato Grosso do Sul state, central-western Brazil. Figure S6: *Amblyomma sculptum* male collected from a ring-tailed coati (*Nasua nasua*) sampled in Campo Grande city, Mato Grosso do Sul state, central-western Brazil. Figure S7: *Amblyomma sculptum* non- engorged female sampled in a ring-tailed coati sampled (*Nasua nasua*) in Campo Grande city, Mato Grosso do Sul state, central-western Brazil. Figure S8: Photographic image of a capybara (*Hydrochoerus hydrochaeris*) (red arrow) near a coati (*Nasua nasua*) in a trap (yellow arrow). Table S1: Values of Odds ratio, Confidence interval (inferior and superior) and *p*-value obtained in the analysis on prevalence of ticks on coatis (*Nasua nasua*) sampled from March 2018 to April 2019 in Campo Grande city, Mato Grosso do sul state, Brazil. Table S2: *p*-value obtained in the analysis on quantity of ticks (*Amblyomma* sp. larvae, *Amblyomma dubitatum* and *Amblyomma sculptum* nymphs) and the interactions between variables locality, sex and age.

## References

[1] Alves-Costa C.P., Da Fonseca G.A.B., Christofaro C. (2004). Variation in the diet of the brown-nosed coati (*Nasua nasua*) in Southeastern Brazil. J. Mammal..

[2] Emmons L., Helgen K. (2016). *Nasua nasua* (Linnaeus, 1766). The IUCN Red List of Threatened Species 2016.

[3] Rodrigues A.F.S.F., Daemon E., Massard C.L. (2006). Ectoparasites of *Nasua nasua* (Carnivora, Procyonidae) from an urban forest in Southeastern Brazil. Arq. Bras. Med. Vet. Zoo.

[4] Cleaveland S., Laurenson M.K., Taylor L.H. (2001). Diseases of humans and their domestic mammals: Pathogen characteristics: Host and the risk of emergence. Philos. Trans. R. Soc. Lond. Biol. Sci..

[5] Otranto D., Cantacessi C., Pfeffer M., Dantas-Torres F., Brianti E., Deplazes P., Genchi C., Guberti V., Capelli G. (2015). The role of wild canids and felids in spreading parasites to dogs and cats in Europe: Part I: Protozoa and tick-borne agents. Vet. Parasitol..

[6] André M.R. (2018). Diversity of *Anaplasma* and *Ehrlichia/Neoehrlichia* Agents in Terrestrial Wild Carnivores Worldwide: Implications for Human and Domestic Animal Health and Wildlife Conservation. Front. Vet. Sci..

[7] Sousa K.C.M., Fernandes M.P., Herrera H.M., Freschi C.R., Machado R.Z., André M. (2017). Diversity of piroplasmids among wild and domestic mammals and ectoparasites in Pantanal wetland, Brazil. Ticks Tick Borne Dis..

[8] Sousa K.C.M., Calchi A.C., Herrera H.M., Dumler J.S., Barros-Battesti D.M., Machado R.Z., André M. (2017). Anaplasmataceae agents among wild mammals and ectoparasites in Brazil. Epidemiol. Infect..

[9] Sousa K.C.M., Herrera H.M., Secato C.T., Oliveira A.D.V., Santos F.M., Rocha F.L., Barreto W.T.G., Macedo G.C., de Andrade Pinto P.C.E., Machado R.Z. (2017). Occurrence and molecular characterization of hemoplasmas in domestic dogs and wild mammals in a Brazilian wetland. Acta Trop..

[10] Magalhães-Matos P.C., Araújo I.M., Valim J.R.A., Ogrzewalska M., Guterres ACordeiro M.D., Cepeda M.C., Fonseca A.H. (2022). Detection of *Rickettsia* spp. in ring-tailed coatis (*Nasua nasua*) and ticks of the Iguaçu National Park, Brazilian Atlantic Rainforest. Ticks Tick Borne Dis..

[11] Dantas-Torres F., Martins T.F., Muñoz-Leal S., Onofrio V.C., Barros-Battesti D.M. (2019). Ticks (Ixodida: Argasidae, Ixodidae) of Brazil: Updated species checklist and taxonomic keys. Ticks Tick Borne Dis..

[12] Martins T.F., Luz H.R., MuñozLeal S., Ramirez D.G., Milanelo L., Marques S., Sanches T.C., Onofrio V.C., Acostam I.C.L., Benatti H.R. (2019). A new species of *Amblyomma* (Acari: Ixodidae) associated with monkeys and passerines of the Atlantic rainforest biome, Southeastern Brazil. Ticks Tick Borne Dis..

[13] Muñoz-Leal S., Martins M.M., Nava S., Landulfo G.A., Simons S.M., Rodrigues V.S., Ramos V.N., Suzin A., Szabó M.P.J., Labruna M.B. (2020). *Ornithodoros cerradoensis* n. sp. (Acari: Argasidae), a member of the *Ornithodoros talaje* (Guérin-Méneville, 1849) group, parasite of rodents in the Brazilian Savannah. Ticks Tick Borne Dis..

[14] Onofrio V.C., Guglielmone A.A., Barros-Battesti D.M., Gianizella S.L., Marcili A., Quadros R.M., Marques S., Labruna M.B. (2020). Description of a new species of *Ixodes* (Acari: Ixodidae) and first report of *Ixodes lasallei* and *Ixodes bocatorensis* in Brazil. Ticks Tick Borne Dis..

[15] Krawczak F.S., Martins T.F., Oliveira C.S., Binder L.C., Costa F.B., Nunes P.H., Gregori F., Labruna M.B. (2015). *Amblyomma yucumense* n. sp. (Acari: Ixodidae), a parasite of wild mammals in Southern Brazil. J. Med. Entomol..

[16] Aragão H.B. (1992). Contribuição para a sistemática e biologia dos ixodidas. Partenogênese em carrapatos *Amblyomma agamum* n. sp.. Mem. Inst. Oswaldo Cruz.

[17] Rodrigues D.S., Maciel R., Cunha L.M., Leite R.C., Oliveira P.R. (2010). *Amblyomma rotundatum* (Koch, 1844) (Acari: Ixodidae) two-host life-cycle on Viperidae snake. Rev. Bras. Parasitol. Vet..

[18] Balashov Y.S. (1972). Bloodsuking ticks (Ixodoidea)—Vectors of diseases of man and animals. Miscell. Publ. Entom. Soc. Am..

[19] Guglielmone A.A., Mangold A.J., Aguirre D.H., Gaido A.B. (1990). Ecological aspects of four species of ticks found on cattle, in Salta, Northwest Argentina. Vet. Parasitol..

[20] Labruna M.B., Kasai N., Ferreira F., Faccini J.L.H., Gennari S.M. (2002). Seasonal dynamics of ticks (Acari: Ixodidae) on horses in the state of Sao Paulo, Brazil. Vet. Parasitol..

[21] Oliveira P.R., Borges L.M.F., Leite R.C., Freitas C.M.V. (2003). Seasonal dynamics of the Cayenne tick, *Amblyomma cajennense* on horses in Brazil. Med. Vet. Entomol..

[22] Nava S., Mangold A.J., Guglielmone A.A. (2008). Aspects of the life cycle of *Amblyomma parvum* (Acari: Ixodidae) under natural conditions. Vet. Parasitol..

[23] Guglielmone A.A., Beati L., Barros-Battesti D.M., Labruna M.B., Nava S., Venzal J.M., Mangold A.J., Szabó M.P.J., Martins J.R., González-Acuna D. (2006). Ticks (Ixodidae) on humans in South America. Exp. Appl. Acarol..

[24] Ramos V.N., Osava C.F., Piovezan U., Szabó M.P.J. (2017). Ambush behavior of the tick *Amblyomma sculptum* (*Amblyomma cajennense* complex) (Acari: Ixodidae) in the Brazilian Pantanal. Ticks Tick Borne Dis..

[25] Szabó M.P.J., Labruna M.B., Garcia M.V., Pinter A., Castagnolli K.C., Pacheco R.C., Castro M.B., Veronez V.A., Magalhães G.M., Vogliotti A. (2009). Ecological aspects of the free-living ticks (Acari: Ixodidae) on animal trails within Atlantic rainforest in South–Eastern Brazil. Ann. Trop. Med. Parasitol..

[26] Szabó M.P.J., Martins T.F., Nieri-Bastos F.A., Spolidorio M.G., Labruna M.B. (2012). A surrogate life cycle of *Amblyomma ovale* Koch, 1844. Ticks Tick Borne Dis..

[27] Barros-Battesti D.M., Baggio D. (1992). Ectoparasites Ixodida Leach, 1817 on wild mammals in the State of Paraná, Brazil. Mem. Inst. Oswaldo Cruz.

[28] Figueiredo L.T.M., Badra S.J., Pereira L.E., Szabó M.P.J. (1999). Report on ticks collected in the Southeast and Mid-West regions of Brazil: Analyzing the potential transmission of tick-borne pathogens to man. Rev. Soc. Bras. Med. Trop..

[29] Labruna M.B., Jorge R.S.P., Sana D.A., Ja’como A.T.A., Kashivakura C.K., Furtado M.M., Ferro C., Perez S.A., Silveira L., Santos T.S. (2005). Ticks (Acari: Ixodida) on wild carnivores in Brazil. Exp. Appl. Acarol..

[30] Martins T.F., Milanelo L., Krawczak F.S., Furuya H.R., Fitorra L.S., Dores F.T., Pedro V.S., Hippolito A.G., Labruna M.B. (2017). Diversity of ticks in the wildlife screening center of São Paulo city, Brazil. Cienc. Rural.

[31] Dantas-Torres F., Ferreira D.R.A., de Melo L.M., Lima P.-A.C.P., Siqueira D.B., Rameh-de-Albuquerque L.C., de Melo A.V., Ramos J.A.C. (2010). Ticks on captive and free-living wild animals in northeastern Brazil. Exp. Appl Acarol..

[32] Estevam L.G.T.M., Junior A.A.F., Silvestre B.T., Hemetrio N.S., Almeida L.R., Oliveira M.M., Silva S.M., Ribeiro M.F.B., Silveira J.A.G. (2020). Seven years of evaluation of ectoparasites and vector-borne pathogens among ring-tailed coatis in an urban park in southeastern Brazil. Vet. Parasitol. Reg. Stud. Rep..

[33] Silva M.R.L., Fornazari F., Martins T.F., Hippólito A.G., Rolim L.S., Bisca J.M., Teixeira C.R., O’Dwyer L.H., Weishuhn L.L., Galvin T.J. (2018). A survey of hemoparasites and ectoparasites in *Nasua nasua* Linnaeus, 1766 with a redescription of *Hepatozoon procyonis* Richards, 1961 based on morphological and molecular data. Parasitol. Res..

[34] Barreto W.T.G., Herrera H.M., de Macedo G.C., Rucco A.C., de Assis W.O., Oliveira-Santos L.G., Porfirio G.E.M.O. (2021). Density and survivorship of the South American coati (*Nasua nasua*) in urban areas in Central–Western Brazil. Hystrix Italian J. Mamm..

[35] Costa E.M.J., Mauro R.A. (2008). Secondary dispersion in coatis’ feces *Nasua nasua* e (Linnaeus, 1766) (Mammalia: Procyonidae) in a fragment of Cerrado, Mato Grosso do Sul, Brazil. Neotrop. Biol. Conserv..

[36] Gonçalves L.R., Paludo G., Bisol T.B., Perles L., de Oliveira L.B., de Oliveira C.M., da Silva T.M.V., Nantes W.A.G., Duarte M.A., Santos F.M. (2021). Molecular detection of piroplasmids in synanthropic rodents, marsupials, and associated ticks from Brazil, with phylogenetic inference of a putative novel *Babesia* sp. from white-eared opossum (*Didelphis albiventris*). Parasitol. Res..

[37] Nantes W.A.G., Santos F.M., de Macedo G.C., Barreto W.T.G., Gonçalves L.R., Rodrigues M.S., Chulli J.V.M., Rucco A.C., Assis W.O., Porfírio G.E.O. (2021). Trypanosomatid species in *Didelphis albiventris* from urban forest fragments. Parasitol Res..

[38] Nantes W.A.G., Barreto W.T.G., Santos F.M., Macedo G.C., Rucco A.C., Assis W.O., Porfírio G.E.O., Andrade G.B., Jansen A.M., Herrera H.M. (2019). The influence of parasitism by *Trypanosoma cruzi* in the hematological parameters of the white ear opossum (*Didelphis albiventris*) from Campo Grande, Mato Grosso do Sul, Brazil. Int. J. Parasitol. Paras. Wildl..

[39] Torres J.M., Ferreira C.M., Anjos E. Morcegos (Mammalia, Chiroptera) ocorrentes no parque estadual do prosa, campo grande-ms, e sua importância na manutenção da unidade de conservação. Proceedings of the Conference: Congresso de Natureza, Turismo e Sustentabilidade.

[40] Olifiers N., Bianchi R.C., D’Andrea P.S., Mourão G., Gompper M.E. (2010). Estimating age of carnivores from the Pantanal region of Brazil. Wildl. Biol..

[41] Martins T.F., Onofrio V.C., Barros-Battesti D.M., Labruna M.B. (2010). Nymphs of the genus *Amblyomma* (Acari Ixodidae) of Brazil: Descriptions, redescriptions, and identification key. Ticks Tick Borne Dis..

[42] Dean A.G., Arner T.G., Sunki G.G., Friedman R., Lantinga M., Sangam S., Zubieta J.C., Sullivan K.M., Brendel K.A., Gao Z. (2011). Epi Info™, a Database and Statistics Program for Public Health Professionals.

[43] Margolis G., Esch G.W., Holmes J.C., Kuris A.M., Schad G.A. (1982). The use of ecological terms in parasitology. J. Parasitol..

[44] Ramos V.N., Lemos F.G., Azevedo F.C., Arrais R.C., Lima C.F.M., Candeias I.Z., Szabó M.J.P. (2020). Wild carnivores, domestic dogs and ticks: Shared parasitism in the Brazilian Cerrado. Parasitology.

[45] Martins T.F., Furtado M.M., Jácomo A.T., Silveira L., Sollman R., Torres N.M., Labruna M.B. (2011). Ticks on free-living wild mammals in the region of Emas National Park, Goiás State, central Brazil. Syst. Appl. Acarol..

[46] Martins T.F., Arrais R.C., Rocha F.L., Santos J.P., May Júnior J.A., Azevedo F.C., Paula R.C., Morato R.G., Rodrigues F.H.G., Labruna M.B. (2015). Carrapatos (Acari: Ixodidae) em mamíferos silvestres do Parque Nacional da Serra da Canastra e arredores, Minas Gerais, Brasil. Ciênc. Rural.

[47] Pereira M.C., Szabó M.P., Bechara G.H., Matushima E.R., Duarte J.M., Rechav Y., Fielden L., Keirans J.E. (2000). Ticks (Acari: Ixodidae) associated with wild animals in the Pantanal region of Brazil. J. Med. Entomol..

[48] Cançado P.H.D., Piranda E.M., Mourão G.M., Faccini J.L.H. (2008). Spatial distribution and impact of cattle-raising on ticks in the Pantanal region of Brazil by using the CO_2_ tick trap. Parasitol. Res..

[49] Martins T.F., Barbieri A.R., Costa F.B., Terassini F.A., Camargo L.M., Peterka C.R., Pacheco R.C., Dias R.A., Nunes P.H., Marcili A. (2016). Geographical distribution of *Amblyomma cajennense* (*sensu lato*) ticks (Parasitiformes: Ixodidae) in Brazil, with description of the nymph of *A. cajennense* (*sensu stricto*). Paras. Vectors.

[50] Scinachi C.A., Takeda A.C.G.G., Mucci L.F., Pinter A. (2017). Association of the occurrence of Brazilian spotted fever and Atlantic rain forest fragmentation in the São Paulo metropolitan region, Brazil. Acta Trop..

[51] Barros-Battesti D.M., Arzua M., Bechara G.H. (2006). Carrapatos de Importância Médico-Veterinária da Região Neotropical: Um Guia Ilustrado para Identificação de Espécies.

[52] Labruna M.B., Pacheco R.C., Ataliba A.C., Szabo M.J.P. (2007). Human parasitism by the capibara tick, *Amblyomma dubitatum* (Acari: Ixodida). Entomol. News.

[53] Horta M.C., Labruna M.B., Pinter A., Linardi P.M., Schumaker T.T.S. (2007). *Rickettsia* infection in five areas of the state of Sao Paulo, Brazil. Mem Inst. Oswaldo Cruz.

[54] Nava S., Venzal J.M., Labruna M.B., Mastropaolo M., González E.M., Mangold A.J., Guglielmone A.A. (2010). Hosts, distribution and genetic divergence (16S rDNA) of *Amblyomma dubitatum* (Acari: Ixodidae). Exp. Appl. Acarol..

[55] Rucco A., Herrera H., Santos F., Porfirio G. (2020). Associação interespecífica entre quatis e capivaras em uma área urbana do Brasil. Bol. Mus. Para. Emílio Goeldi Ciênc. Nat..

[56] Ostfeld R.S., Keesing F. (2000). Biodiversity and disease risk: The case of Lyme disease. Conserv. Biol..

[57] Ogrzewalska M., Uezu A., Jenkins C.N., Labruna M.B. (2011). Effect of Forest Fragmentation on Tick Infestations of Birds and Tick Infection Rates by *Rickettsia* in the Atlantic Forest of Brazil. EcoHealth.

[58] Randolph S.E. (2004). Tick ecology: Processes and patterns behind the epidemiological risk posed by ixodid ticks as vectors. Parasitology.

[59] Deblinger R.D., Wilson M.L., Rimmer D.W., Spielman A. (1993). Reduced abundance of immature *Ixodes dammini* (Acari: Ixodidae) following incremental removal of deer. J. Med. Entomol..

[60] Allan B.F., Keesing F., Ostfeld R.S. (2003). Effect of forest fragmentation on Lyme disease risk. Conserv. Biol..

[61] Coetzer J.A.W., Tustin R.C. (2004). Infectious Diseases of Livestock with Special Reference to Southern Africa.

[62] Madder M., Thys E., Achi L., Toure A., De Deken R. (2011). *Rhipicephalus (Boophilus) microplus:* A most successful invasive tick species in West-Africa. Exp. Appl. Acarol..

[63] Cançado P.H.D., Zucco C.A., Piranda E.M., Faccini J.L.H., Mourão G.M. (2009). *Rhipicephalus (Boophilus) microplus* (Acari: Ixodidae) as a parasite of pampas deer (*Ozoctoceros bezoarticus*) and cattle in Brazil’s Central Pantanal. Rev. Bras. Parasitol. Vet..

[64] Dantas-Torres F. (2008). The brown dog tick, *Rhipicephalus sanguineus* (Latreille, 1806) (Acari: Ixodidae): From taxonomy to control. Vet. Parasitol..

[65] Szabó M.P.J., Pascoli G.V.T., Marçal Júnior O., Franchin A.G., Torga K. (2008). Carrapato vermelho do cão *Rhipicephalus sanguineus* parasitando *Coereba flaveola* no cerrado brasileiro. Cienc. Rural.

[66] Gregory R.D., Montgomery S.S.J., Montgomery W.I. (1992). Population biology of *Heligmosomoides polygyrus* (Nematoda) in the wood mouse. J. Anim. Ecol..

[67] Hudson P.J., Dobson A.P., Clayton D.H., Moore J. (1997). Host-parasite processes and demographic consequences. Host-Parasite Evolution: General Principles and Avian Models.

[68] Hirsch B.T. (2007). Spoiled brats: Is extreme juvenile agonism in ring-tailed coatis (*Nasua nasua*) dominance or tolerated aggression?. Ethology.

[69] Olifiers N., Bianchi R.C., Mourao G.M., Gompper M.E. (2009). Construction of arboreal nests by brown-nosed coatis, *Nasua nasua* (Carnivora: Procyonidae) in the Brazilian Pantanal. Zoologia.

[70] Klein S.L. (2004). Hormonal and immunological mechanisms mediating sex differences in parasite infection. Parasit. Immunol..

[71] Hokan M., Strube C., Radespiel U., Zimmermann E. (2017). Sleeping site ecology, but not sex, affect ecto- and hemoparasite risk, in sympatric, arboreal primates (*Avahi occidentalis* and *Lepilemur edwardsi*). Front. Zool..

[72] Shiddo S.A., Aden Mohamed A., Akuffo H.O., Mohamud K.A., Herzi A.A., Mohamed H.A., Huldt G., Nilsson L.A., Ouchterlony O., Thorstensson R. (1995). Visceral leishmaniasis in Somalia: Prevalence of markers of infection and disease manifestations in a village in an endemic area. Trans. R. Soc. Med. Hyg..

[73] Gittleman J.L., Gittleman J.L. (1989). Carnivore group living: Comparative trends. Carnivore Behavior, Ecology, and Evolution.

[74] Gompper M.E. (1996). Sociality and a sociality in white-nosed coatis (*Nasua narica*): Foraging costs and benefits. Behav. Ecol..

[75] Beisiegel B.M. (2001). Notes on the coati, *Nasua nasua* (Carnivora: Procyonidae) in an Atlantic Forest area. Braz. J. Biol..

