# Supplementary material for: Diversity and Seasonal Dynamics of Ticks on Ring-Tailed Coatis Nasua nasua (Carnivora: Procyonidae) in Two Urban Areas from Midwestern Brazil
Source: Animals (Basel). 2022 Jan 25;12(3):293. doi: 10.3390/ani12030293 (PMC8833741; doi:10.3390/ani12030293)
Supplement: Supplementary file 1 [file animals-12-00293-s001.zip › animals-1530807-supplementary.pdf]

Diversity and Seasonal Dynamics of Ticks on Ring-Tailed Coatis *Nasua nasua* (Carnivora: Procyonidae) in Two Urban Areas from Midwestern Brazil

Livia Perles [1], Thiago Fernandes Martins [2,3], Wanessa Barreto [4], Gabriel Carvalho de Macedo [5], Heitor Herrera [5], Luis Antônio Mathias [6], Marcelo Bahia Labruna [2], Darci Moraes Barros-Battesti [1], Rosangela Zacarias Machado [1], and Marcos Rogério André [1,*]

Supplementary file. Voucher material deposited in the IBSP collection.

Amblyomma **sp.**

10 larvae (IBSP 16757); Parque Estadual do Prosa, Campo Grande Municipality, Mato Grosso do Sul State, Brazil; 02 Aug. 2018; ex. *Nasua nasua* (Linnaeus 1766) (Carnivora: Procyonidae); L. Perles et al. coll.;5 larvae (IBSP 16774); same locality; same host; 11 May 2018; 4 larvae (IBSP 16802A); 30 May. 2018; 6 larvae (IBSP 16806A); same data; 4 larvae (IBSP 16779); 06 Aug. 2018; 2 larvae (IBSP 16801); 13 Mar. 2018; 1 larva (IBSP 16805); same day; 4 larvae (IBSP 16807B); same locality; same host; 31 Jul. 2018; 8 larvae (IBSP 16809B); 15 Jun. 2018; 2 larvae (IBSP 168010B); 30 May 2018; 7 larvae (IBSP 16812A); 15 Mar. 2018; 1 larva (IBSP 16813); 16 Mar. 2018; 1 larva (IBSP 16814A); 16 Mar. 2018; 1 larva (IBSP 16815B); same data; 3 larvae (IBSP 16816A); 19 Mar. 2018; 3 larvae (IBSP 16816A); 19 Mar. 2018; 1 larva (IBSP 16817B); 20 Mar. 2018; 3 larvae (IBSP 16818A); 21 Mar. 2018; 16 larvae (IBSP 16821B); 15. May 2018; 4 larvae (IBSP 16823A); same data; 31 larvae (IBSP 16822B); 22. May 2018; 5 larvae (IBSP 16824); 28. May 2018; 3 larvae (IBSP 16825); same data; 5 larvae (IBSP 16826); 29 May 2018; 3 larvae (IBSP 16827A); same data; 4 larvae (IBSP 16828B); same data; 18 larvae (IBSP 16804A); 30 May. 2019; 7 larvae (IBSP 16829B); 30 May 2018; 19 larvae (IBSP 16830); 12 Jun. 2018; 9 larvae (IBSP 16831); 12 Jun. 2018; 3 larvae (IBSP 16834A); 31 Jul. 2018; 5 larvae (IBSP 16835C); same data; 3 larvae (IBSP 16836A); 01 Aug. 2018; 16 larvae (IBSP 16837A); 02 Aug. 2018; 4 larvae (IBSP 16839A); 03 Aug. 2018; 9 larvae (IBSP 16840B); same data; 3 larvae (IBSP 16842A); 06 Aug. 2018; 4 larvae (IBSP 16844); 08 Aug. 2018; 13 larvae (IBSP 16765); same host; Vila da Base Aérea, Campo Grande Municipality, Mato Grosso do Sul State, Brazil; 23 March 2019; 3 larvae (IBSP 16766B); same host; same locality; 01 Nov. 2018; 15 larvae (IBSP 16767B); same host; same locality; 30 Jan. 2019; 20 larvae (IBSP 16768); same host; same locality; 29 Jan. 2019; 1 larva (IBSP 16771); 26 Jun. 2018; 7 larvae (IBSP 16772); 29 Jan. 2019; 1 larva (IBSP 16773); 23 Jan. 2019; 33 larvae (IBSP 16777); 30 Apr. 2019; 33 larvae (IBSP 16779B); 06 Aug. 2018; 7 larvae (IBSP 16782); 18 May 2018; 1 larva (IBSP 16785A); 24 Aug 2018; 2 larvae (IBSP 16788); 20 May 2018; 1 larva (IBSP 16793); 21 Mar. 2019; 32 larva (IBSP 16797); 25 Mar. 2019; 20 larva (IBSP 16847A); 30 Apr. 2018; 11 larva (IBSP 16848); 02 May. 2018; 3 larvae (IBSP 16850B); 28 Aug. 2018; 15 larvae (IBSP 16852A); 02 May 2018; 2 larvae (IBSP 16855); same data; 5 larvae (IBSP 16856); 03 May 2018.

Amblyomma ovale **(Koch 1844)**

1 ♀ (IBSP 16854); Vila da Base Aérea, Campo Grande Municipality, Mato Grosso do Sul State, Brazil; 21 Aug. 2018; ex. *Nasua nasua* (Linnaeus 1766) (Carnivora: Procyonidae); L. Perles et al. coll. 1 ♀ (IBSP 16860B); Vila da Base Aérea, Campo Grande Municipality, Mato Grosso do Sul State, Brazil; 19 May. 2018; ex. *Nasua nasua* (Linnaeus 1766) (Carnivora: Procyonidae); L. Perles et al. coll. 1 ♀ (IBSP 16863C); Vila da Base Aérea, Campo Grande Municipality, Mato Grosso do Sul State, Brazil; 04 May. 2018; ex. *Nasua nasua* (Linnaeus 1766) (Carnivora: Procyonidae); L.

Perles et al. coll. 1 ♂ (IBSP 16862); Vila da Base Aérea, Campo Grande Municipality, Mato Grosso do Sul State, Brazil; 23 Oct. 2018; ex. *Nasua nasua* (Linnaeus 1766) (Carnivora: Procyonidae); L. Perles et al. coll. 1 ♂ (IBSP 16769A); Vila da Base Aérea, Campo Grande Municipality, Mato Grosso do Sul State, Brazil; 20 Aug. 2018; ex. *Nasua nasua* (Linnaeus 1766) (Carnivora: Procyonidae); L. Perles et al. coll. 1 ♀ (IBSP 16864); Vila da Base Aérea, Campo Grande Municipality, Mato Grosso do Sul State, Brazil; 04 May 2018; ex. *Nasua nasua* (Linnaeus 1766) (Carnivora: Procyonidae).

***Amblyomma dubitatum* (Neumann 1899)**

1 nymphae (IBSP 16749); Vila da Base Aérea, Campo Grande Municipality, Mato Grosso do Sul State, Brazil; 23 Jan. 2018; ex. *Nasua nasua* (Linnaeus 1766) (Carnivora: Procyonidae); L. Perles et al. coll. 5 nymphs (IBSP 16750); same data; 2 nymphae (IBSP 16754); same locality and host; 30 April 2019; 1 nymphae (IBSP 16758); same locality; same host; 22 Jan. 2019; 1 nymphae (IBSP 16759A); same locality; same host; 06 Nov. 2018; 1 nymphae (IBSP 16764); same locality; same host; 30 Aug. 2018; 1 nymph (IBSP 16767A); same locality; same host; 30 Jan. 2019; 1 nymphae (IBSP 16770); same locality; same host; 23 Apr. 2019; 1 nymphae (IBSP 16776); same locality; 29 Jan. 2019; same host; 1 nymphae (IBSP 16780B); same locality; 23 Jan. 2019; 1 nymphae (IBSP 16786); 30 Jan. 2019; 1 nymphae (IBSP 16787); 31 Out. 2018; 1 nymphae (IBSP 16798); same locality; same host; 24 Aug. 2018; 1 nymphae (IBSP 16753B); Parque Estadual do Prosa, Campo Grande Municipality, Mato Grosso do Sul State, Brazil; 04 Out. 2018; ex. *Nasua nasua* (Linnaeus 1766) (Carnivora: Procyonidae); L. Perles et al. coll. 1 nymphae (IBSP 16800); 31 July 2018; 1 nymphae (IBSP 16803B); 13 Mar. 2018; 1 nymphae (IBSP 16806C); Parque Estadual do Prosa, Campo Grande Municipality, Mato Grosso do Sul State, Brazil; 30 May. 2018; ex. *Nasua nasua* (Linnaeus 1766) (Carnivora: Procyonidae); L. Perles et al. coll. 1 nymphae (IBSP 16808B); 14 Mar. 2018; 1 nymphae (IBSP 16812B); 15 Mar. 2018; 1 nymphae (IBSP 16816B); 19 Mar. 2018; 1 nymphae (IBSP 16818B); 21 Mar. 2018; 1 nymphae (IBSP 16819); 21 Mar. 2018; 1 nymphae (IBSP 16827B); Parque Estadual do Prosa, Campo Grande Municipality, Mato Grosso do Sul State, Brazil; 29 May. 2018; ex. *Nasua nasua* (Linnaeus 1766) (Carnivora: Procyonidae); L. Perles et al. coll. 1 nymphae (IBSP 16832A); Parque Estadual do Prosa, Campo Grande Municipality, Mato Grosso do Sul State, Brazil; 31 Jul. 2018; ex. *Nasua nasua* (Linnaeus 1766) (Carnivora: Procyonidae); L. Perles et al. coll. 1 nymphae (IBSP 16833A); Parque Estadual do Prosa, Campo Grande Municipality, Mato Grosso do Sul State, Brazil; 31 Jul. 2018; ex. *Nasua nasua* (Linnaeus 1766) (Carnivora: Procyonidae); L. Perles et al. coll. 1 nymphae (IBSP 16835A); Parque Estadual do Prosa, Campo Grande Municipality, Mato Grosso do Sul State, Brazil; 31 Jul. 2018; ex. *Nasua nasua* (Linnaeus 1766) (Carnivora: Procyonidae); L. Perles et al. coll. 1 nymphae (IBSP 16836B); 01 Aug. 2018; 1 nymphae (IBSP 16838A); 02 Aug. 2018; 1 nymphae (IBSP 16839B); Parque Estadual do Prosa, Campo Grande Municipality, Mato Grosso do Sul State, Brazil; 03 Aug. 2018; ex. *Nasua nasua* (Linnaeus 1766) (Carnivora: Procyonidae); L. Perles et al. coll. 1 nymphae (IBSP 16842B); Parque Estadual do Prosa, Campo Grande Municipality, Mato Grosso do Sul State, Brazil; 06 Aug. 2018; ex. *Nasua nasua* (Linnaeus 1766) (Carnivora: Procyonidae); L. Perles et al. coll. 1 nymph (IBSP 16853C); Vila da Base Aérea, Campo Grande Municipality, Mato Grosso do Sul State, Brazil; 02 May. 2018; ex. *Nasua nasua* (Linnaeus 1766) (Carnivora: Procyonidae); L. Perles et al. coll. 1 nymphae (IBSP 16863B); Vila da Base Aérea, Campo Grande Municipality, Mato Grosso do Sul State, Brazil; 04 May. 2018; ex. *Nasua nasua* (Linnaeus 1766) (Carnivora:

Procyonidae); L. Perles et al. coll. 1 nymphae (IBSP 16878A); Vila da Base Aérea, Campo Grande Municipality, Mato Grosso do Sul State, Brazil; 24 Aug. 2018; 1 nymphae (IBSP 16881); Vila da Base Aérea, Campo Grande Municipality, Mato Grosso do Sul State, Brazil; 31 Out. 2018; ex. *Nasua nasua* (Linnaeus 1766) (Carnivora: Procyonidae); L. Perles et al. coll. 1 nymphae (IBSP 16882); 31 Aug. 2018.

Amblyomma sculptum (Berlese 1888)

4 nymphae (IBSP 16802B); Parque Estadual do Prosa, Campo Grande Municipality, Mato Grosso do Sul State, Brazil; 30 May. 2018; ex. *Nasua nasua* (Linnaeus 1766) (Carnivora: Procyonidae); L. Perles et al. coll.; 7 nymphae (IBSP 16804B); same data; 18 nymphae (IBSP 16792); same locality; same host; 8 Oct. 2018; 1 nymph (IBSP 16803A); 13 Mar. 2018; 4 nymphae (IBSP 16806B); same locality; same host; 30 May 2018; 9 nymphae (IBSP 16807A); same locality; same host; 31 Jul. 2018; 1 nymph (IBSP 16808A); same locality; same host; 14 Mar. 2018; 3 nymphae (IBSP 16809A); same locality; same host; 15 Jun. 2018; 9 nymphae (IBSP 16790); same locality; same host; 30 Jul. 2018; 10 nymphae (IBSP 16810A); same locality; same host; 30 May 2018; 11 nymphae (IBSP 16755); same locality; same host; 1 Aug. 2018; 1 nymph (IBSP 16814B); same locality; same host; 16 Mar. 2018; 2 nymphae (IBSP 16815A); same data; 1 nymph (IBSP 16817A); same locality; same host; 20 Mar. 2018; 2 nymphae (IBSP 16818C); same locality; same host; 21 Mar. 2018; 2 nymphae (IBSP 16820B); same locality; same host; 22 Mar. 2018; 4 nymphae (IBSP 16822A); same data; 2 nymphae (IBSP 16821A); same locality; same host; 15 Jun. 2018; 7 nymphae (IBSP 16823B); same data; 17 nymphae (IBSP 16762); same locality; same host; 1 Oct. 2018; 1 nymph (IBSP 16828A); same locality; same host; 29 May 2018; 12 nymphae (IBSP 16829A); same locality; same host; 30 May 2018; 30 nymphae (IBSP 16781); same locality; same host; 30 Jul. 2018; 8 nymphae (IBSP 16832B); same locality; same host; 31 Jul. 2018; 6 nymphae (IBSP 16833B); same data; 4 nymphae (IBSP 16834B); same data; 7 nymphae (IBSP 16835B); same data; 7 nymphae (IBSP 16836C); same locality; same host; 01 Aug. 2018; 6 nymphae (IBSP 16837B); same locality; same host; 02 Aug. 2018; 8 nymphae (IBSP 16838B); same data; 11 nymphae (IBSP 16799); same locality; same host; 04 Oct. 2018; 10 nymphae (IBSP 16839C); same locality; same host; 31 Jul. 2018; 11 nymphae (IBSP 16840); same locality; same host; 03 Aug. 2018; 17 nymphae (IBSP 16841); same locality; same host; 03 Aug. 2018; 15 nymphae (IBSP 16779A); same locality; same host; 06 Aug. 2018; 7 nymphae (IBSP 16843); same locality; same host; 07 Aug. 2018; 4 nymphae (IBSP 16844); same locality; same host; 08 Aug. 2018; 17 nymphae (IBSP 16845); same data; 2 nymphae (IBSP 16847B); Vila da Base Aérea, Campo Grande Municipality, Mato Grosso do Sul State, Brazil; ex. *Nasua nasua* (Linnaeus 1766) (Carnivora: Procyonidae); 30 Apr. 2018; L. Perles et al. coll. 7 nymphae (IBSP 16849); same locality; same host; 18 Jun. 2018; 9 nymphae (IBSP 16851); same locality; same host; 23 Oct. 2018; 2 nymphae (IBSP 16852B); same locality; same host; 1 nymph (IBSP 16853B); same data; 19 nymphae (IBSP 16761); same locality; same host; 22 Oct. 2018; 8 nymphae (IBSP 16860A); same locality; same host; 19 Jun. 2018; 1 nymph (IBSP 16861B); same locality; same host; 4 May 2018; 1 nymph (IBSP 16863B); same data; 1 nymph (IBSP 16867A); same locality; same host; 10 May 2018; 2 nymphae (IBSP 16847B); same locality; same host; 33 nymphae (IBSP 16752); same locality; same host; 31 Aug. 2018; 27 nymphae (IBSP 16785B); same locality; same host; 24 Aug. 2018; 4 nymphae (IBSP 16869B); same locality; same host; 21 Jun. 2018; 4 nymphae (IBSP 16870); same locality; same host; 22 Jun. 2018; 13 nymphae (IBSP 16871); same locality; same host; 22

Jun. 2018; 12 nymphae (IBSP 16872B); same locality; same host; 24 Aug. 2018; 1 nymph (IBSP 16873); same locality; same host; 21 Mar. 2018; 11 nymphae (IBSP 16875); same locality; same host; 26 Jun. 2018; 9 nymphae (IBSP 16875B); same locality; same host; 26 Jun. 2018; 14 nymphae (IBSP 16760); same locality; same host; 06 Nov. 2018.

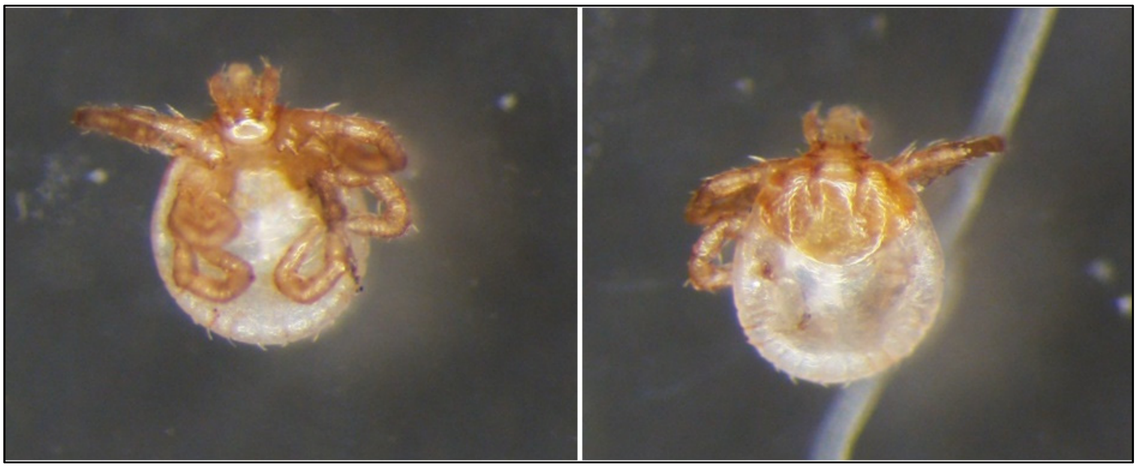

Figure S1. Non-engorged *Amblyomma* spp. larvae collected from a ring-tailed coati (*Nasua nasua*) sampled in Campo Grande city, Mato Grosso do Sul state, central-western Brazil. A) Ventral view. B) Dorsal view.

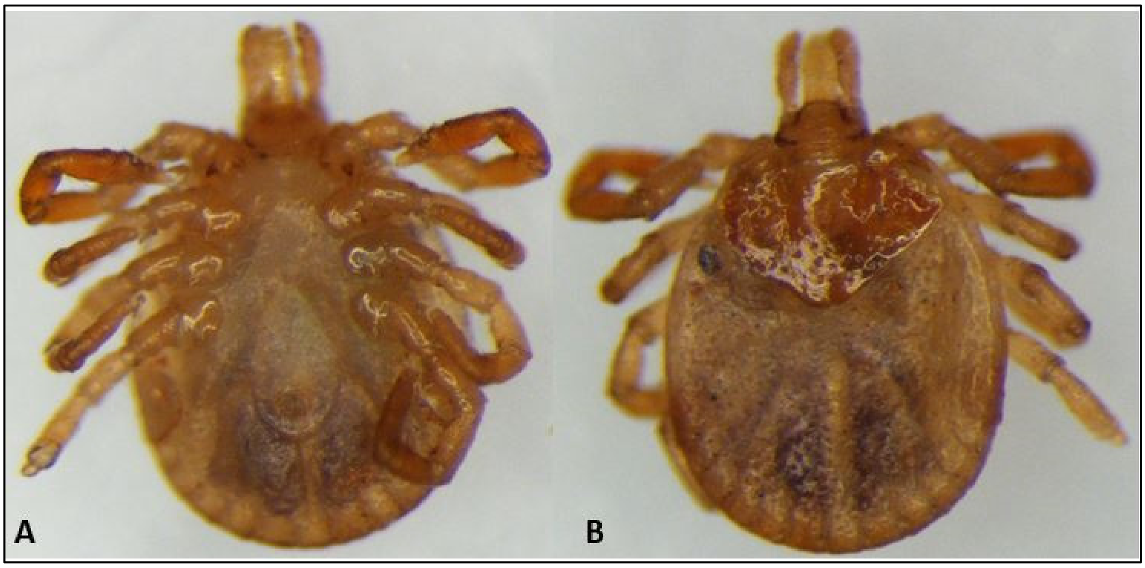

Figure S2. *Amblyomma sculptum* nymphs collected from ring-tailed coatis (*Nasua nasua*) sampled in Campo Grande city, Mato Grosso do Sul state, central-western Brazil. A) Ventral view. B) Dorsal view. GenBank acession number: MT974144.

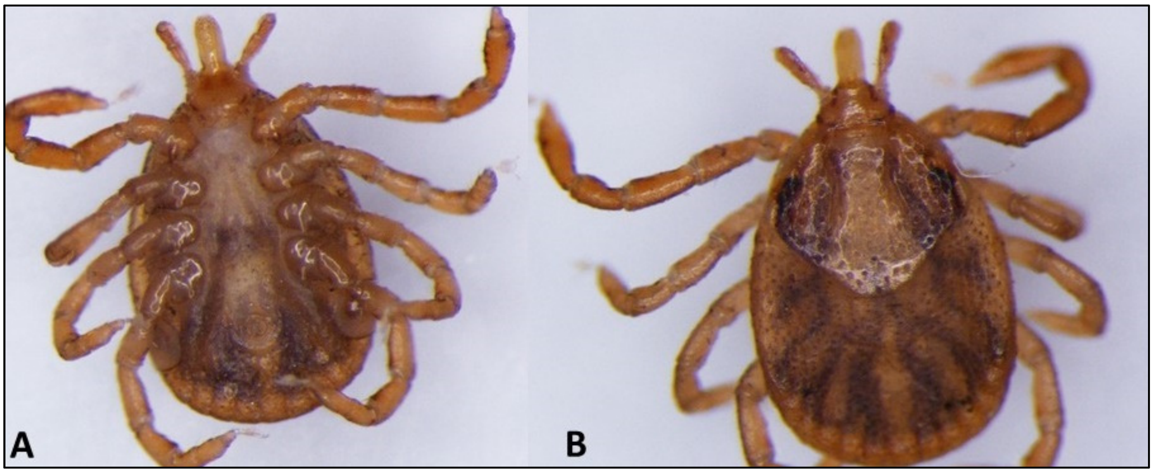

Figure S3. *Amblyomma dubitatum* nymphs collected from ring-tailed coatis (*Nasua nasua*) sampled in Campo Grande city, Mato Grosso do Sul state, central-western Brazil. A) Ventral view. B) Dorsal view. GenBank acession number: MT974145.

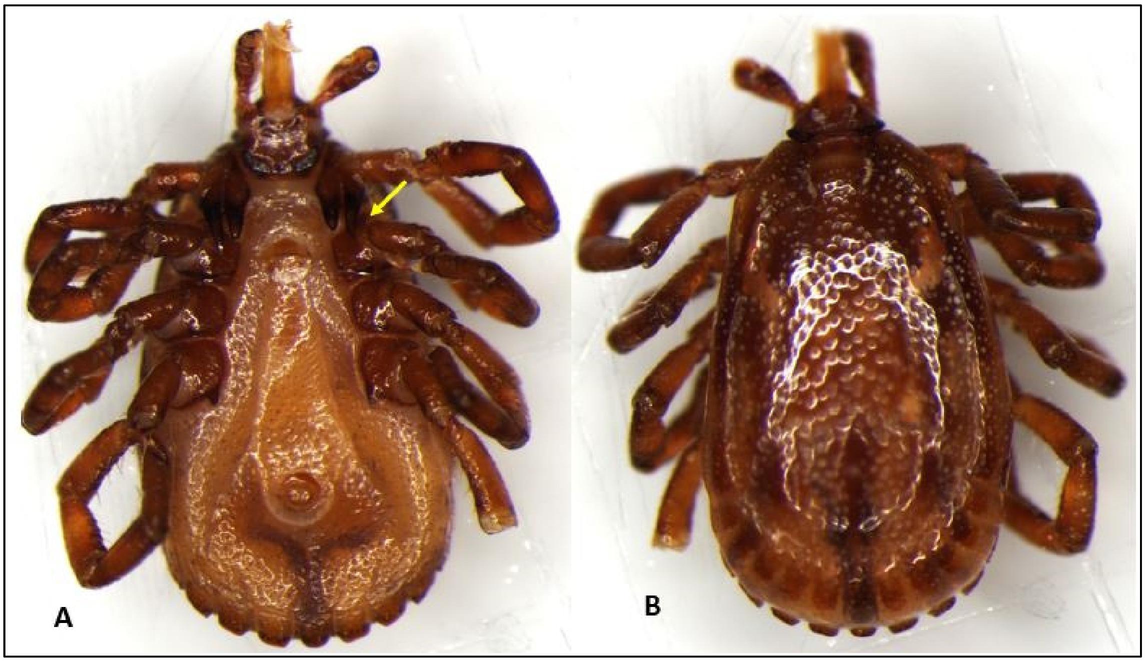

Figure S4. *Amblyomma ovale* male collected from a ring-tailed coati (*Nasua nasua*) in Campo Grande city, Mato Grosso do Sul state, central-western Brazil. A) Ventral view: two strong spines can be seen on thigh 1, with the outside spine turned slightly outwards (arrow). B) Dorsal view.

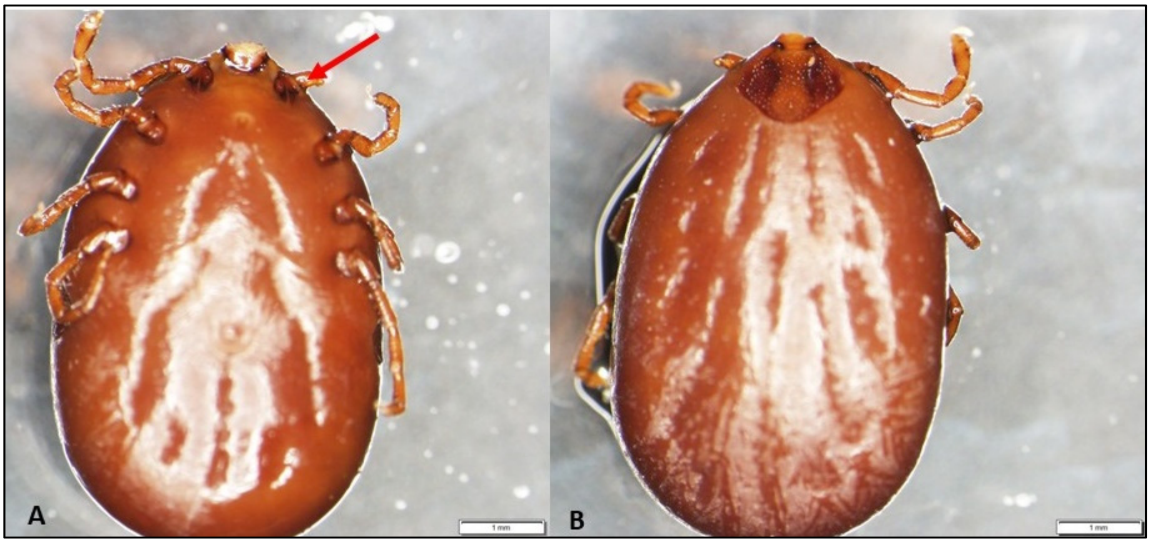

Figure S5. *Amblyomma ovale* engorged female collected from a ring-tailed coati (*Nasua nasua*) sampled in Campo Grande city, Mato Grosso do Sul state, central-western Brazil. A) Ventral view: two strong spines can be seen on thigh 1, with the outside spine turned slightly outwards (arrow). B) Dorsal view.

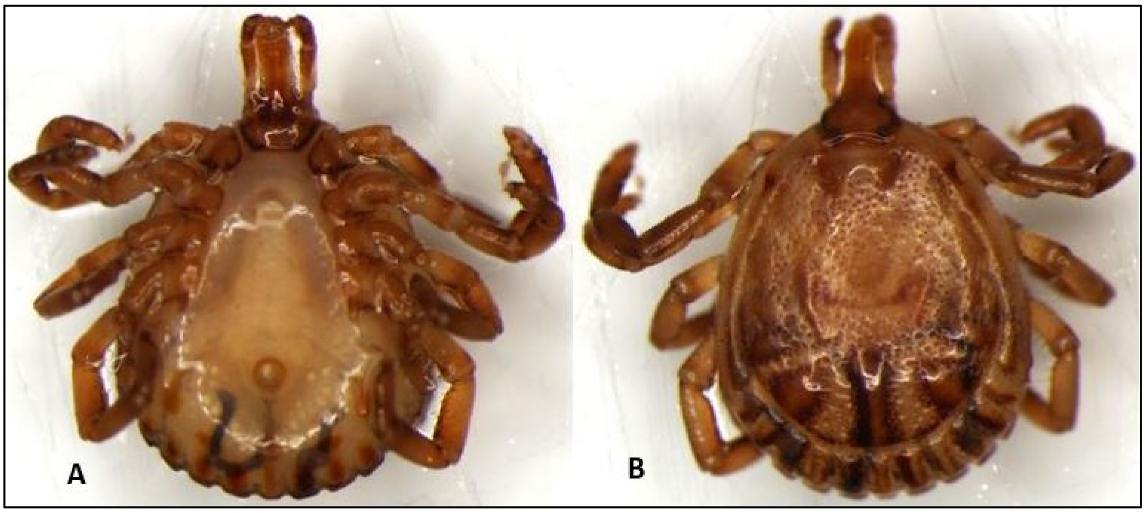

Figure S6. *Amblyomma sculptum* male collected from a ring-tailed coati (*Nasua nasua*) sampled in Campo Grande city, Mato Grosso do Sul state, central-western Brazil. A) Ventral view. B) Dorsal view.

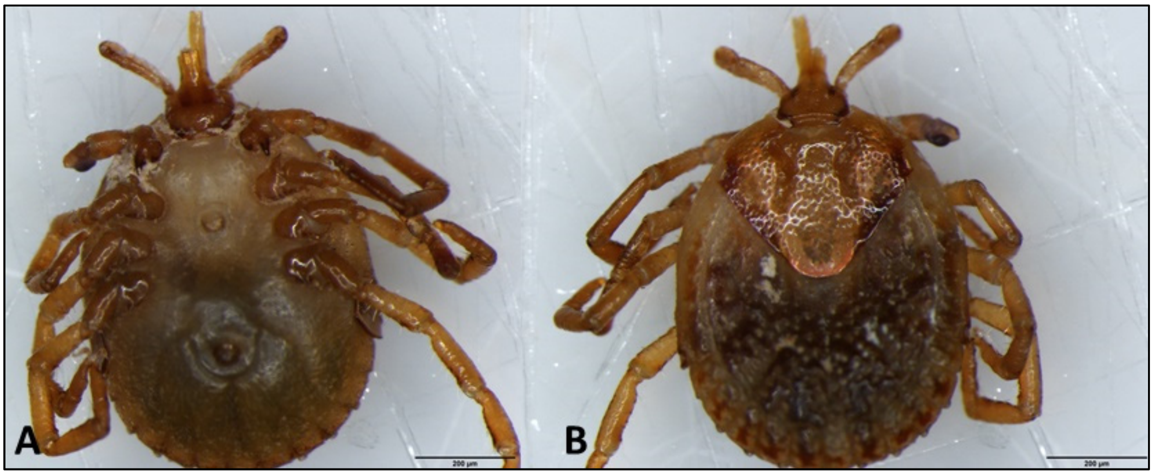

Figure S7. *Amblyomma sculptum* non- engorged female sampled in a ring-tailed coati sampled (*Nasua nasua*) in Campo Grande city, Mato Grosso do Sul state, central-western Brazil. A) Ventral view. B) Dorsal view.

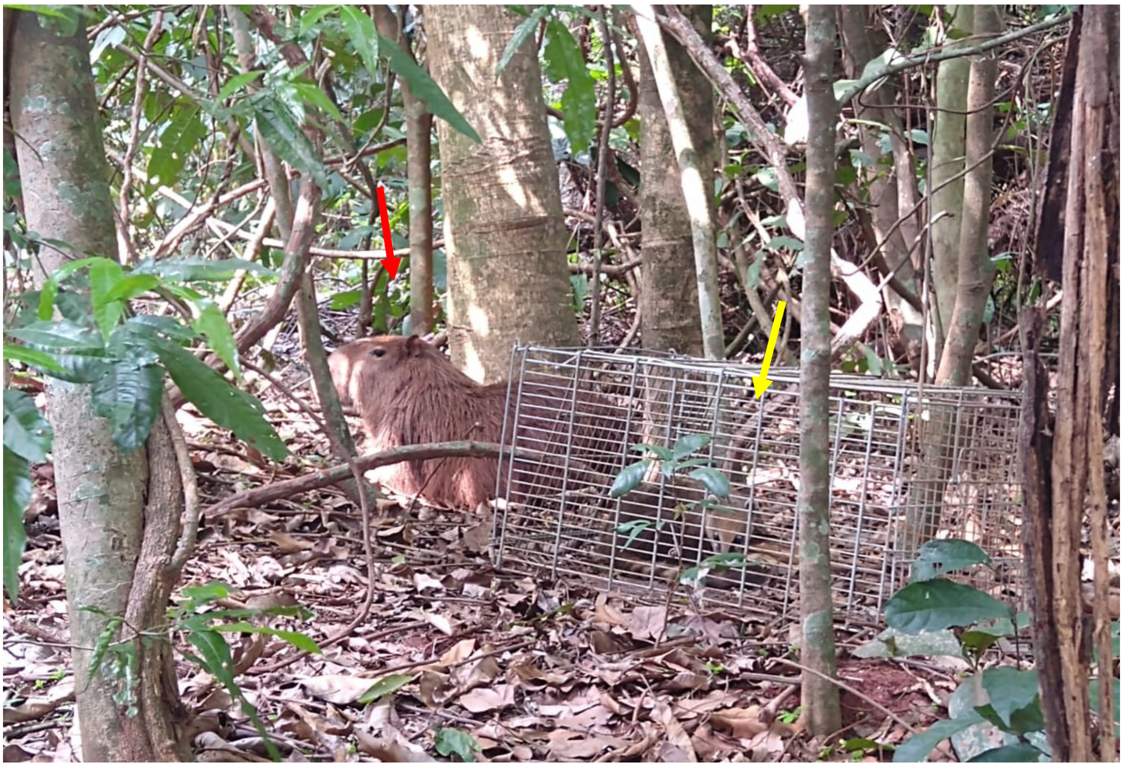

Figure S8. Photographic image of a capybara (*Hydrochoerus hydrochaeris*) (red arrow) near a coati (*Nasua nasua)* in a trap (yellow arrow).

Table S1. Values of Odds ratio, Confidence interval (inferior and superior) and *p*-value obtained in the analysis on prevalence of ticks on coatis (*Nasua nasua*) sampled from March 2018 to April 2019 in Campo Grande city, Mato Grosso do sul state, Brazil.

Categorical Independent Variables		*Amblyomma* sp. larvae		OR	ICinf	ICsup	*p-value*
		Presence	Absence				
Locality	PEP	66.67% (32/48)	33.33% (16/48)				
	VBA	56.36% (31/55)	43.64% (24/55)	0.64	0.28	1.44	0.38
Sex	F	68.25% (43/63)	31.75% (20/63)				
	M	50% (20/40)	50% (20/40)	0.46	0.20	1.05	0.09
Age	Immature	46.15% (12/26)	53.85% (14/26)				
	Mature	66.23% (51/77)	33.77% (26/77)	22.88	0.92	5.65	0.11
**Categorical Independent Variables**		***Amblyomma dubitatum* nymphs**		**OR**	**ICinf**	**ICsup**	***p-value***
Locality	PEP	47.92% (23/48)	52.08% (25/48)				
	VBA	38.18% (21/55)	61.82% (34/55)	0.67	0.30	1.47	0.42
Sex	F	41.27% (26/63)	58.73% (37/63)				
	M	45% (18/40)	55% (22/40)	11.46	0.52	2.59	0.86
Age	Immature	46.15% (12/26)	53.85% (14/26)				
	Mature	41.56% (32/77)	58.44% (45/77)	0.82	0.33	2.02	0.85
**Categorical Independent Variables**		***Amblyomma* sculptum nymphs**		**OR**	**ICinf**	**ICsup**	***p-value***
Locality	PEP	75.00% (36/48)	15.00% (12/48)				
	VBA	76.36% (42/55)	23.64% (13/55)	10.76	0.43	2.65	1
Sex	F	73.02% (46/63)	26.98% (17/63)				
	M	80.00% (32/40)	20.00% (8/40)	1.47	0.54	3.84	1
Age	Immature	76.92% (20/26)	23.08% (6/26)				
	Mature	75.32% (58/77)	24.68% (19/77)	0.91	0.32	2.58	1

OR: *Odds Ratio* ; **ICinf**: Inferior confidence interval; **ICsup**: Superior confidence interval; **F**: Female; **M**: Male; **PEP**: Parque Estadual do Prosa; **VBA**: Vila da Base Aérea

Table S2. *P*-value obtained in the analysis on quantity of ticks (*Amblyomma* sp. larvae, *Amblyomma dubitatum* and *Amblyomma sculptum* nymphs) and the interactions between variables locality, sex and age.

Variable	*p*-value		
	Amblyomma sp. larvae	*Amblyomma dubitatum* nymphs	*Amblyomma sculptum* nymphs
Locality	0.29	0.21	0.35
Sex	0.94	0.83	0.76
Locality and sex	0.47	0.62	0.83
Age	0.51	0.97	0.30
Locality and age	0.77	0.17	0.40
Sex and age	0.63	0.46	0.21
Locality. sex and age	0.74	0.97	0.48
